# The ablation margin as a regenerative and immunosuppressive niche in hepatocellular carcinoma: multicellular remodeling and organoid-on-a-chip modeling

**DOI:** 10.3389/fbioe.2026.1952704

**Published:** 2026-09-29

**Authors:** Tingyue Qiao, Yang Zha, Honghua Dai, Yue Chai, Li Weng

**Affiliations:** 1 Department of Hepatobiliary Surgery, Chongqing Qijiang District People’s Hospital (Jiangsu Provincial People’s Hospital Chongqing Hospital), Chongqing, China; 2 Breast Disease Center, The First Affiliated Hospital with Nanjing Medical University, Nanjing, Jiangsu, China; 3 Hepatobiliary Center, The First Affiliated Hospital of Nanjing Medical University, Key Laboratory of Liver Transplantation, Chinese Academy of Medical Sciences, NHC Key Laboratory of Liver Transplantation, Nanjing, Jiangsu, China; 4 Department of Endocrinology, Chongqing Qijiang District People’s Hospital (Jiangsu Provincial People’s Hospital Chongqing Hospital), Chongqing, China

**Keywords:** extracellular matrix remodeling, hepatocellular carcinoma, local tumor progression, organoid-on-a-chip, thermal ablation, tumor microenvironment

## Abstract

Thermal ablation is an important local treatment for hepatocellular carcinoma (HCC), yet post-ablation local tumor progression remains a major obstacle to durable disease control. Traditionally, recurrence has been attributed mainly to incomplete thermal coverage, inadequate ablative margins, or heat-sink effects. However, increasing evidence suggests that the sublethal thermal injury (STI) zone at the ablation margin is not merely a passive physical boundary, but a biologically active injury-repair microenvironment. Within this region, gradients of thermal dose, hypoxia, damage-associated molecular patterns (DAMPs), extracellular matrix mechanics, and immune infiltration may interact to shape residual tumor cell behavior. Post-ablation remodeling is likely driven by coordinated crosstalk among myeloid cells, hepatic stellate cells/cancer-associated fibroblast-like stromal cells (HSCs/CAFs), endothelial cells, neutrophils, adaptive immune cells, and residual tumor cells. These interactions may promote epithelial–mesenchymal transition, stem-like plasticity, immune evasion, angiogenic repair, and local tumor progression. Current two-dimensional (2D) cultures lack three-dimensional architecture and multicellular spatial interactions, whereas animal models are limited by variable thermal dose control, restricted real-time imaging, and interspecies differences. Emerging HCC organoid-on-a-chip platforms may help address these gaps by integrating patient-derived organoids (PDOs), tunable extracellular matrix hydrogels, microfluidic thermal and hypoxic gradients, and defined multicellular co-culture modules. Importantly, immune checkpoint testing requires immune-competent extensions incorporating autologous T cells, and antigen-presentation studies require dendritic cells. This review discusses the ablation margin as a multicellular recurrence niche and evaluates organoid-on-a-chip systems as hypothesis-generating tools for mechanism-guided and safety-conscious adjuvant strategy development.

## 1 Introduction

Thermal ablation, including radiofrequency ablation and microwave ablation, is a standard local treatment for hepatocellular carcinoma (HCC), particularly for patients with early-stage tumors or those who are not suitable candidates for surgical resection or transplantation (European Association for the Study of the Liver, 2025). Despite improvements in imaging guidance, probe design, and treatment planning, local tumor progression after apparently successful ablation remains a clinically important cause of treatment failure and impaired long-term survival (Tanaka et al., 2023; Hu et al., 2025). Traditionally, marginal recurrence has been attributed mainly to incomplete thermal coverage, insufficient safety margins, or the heat-sink effect caused by adjacent vessels (Pillai et al., 2015; Chu and Dupuy, 2014). These technical factors are undoubtedly important; however, they do not fully explain rapid tumor regrowth in some patients whose post-procedural imaging suggests complete ablation with adequate margins. This discrepancy indicates that biological remodeling at the ablation margin may also contribute to post-ablation recurrence (Ahmed et al., 2011).

Thermal ablation produces a spatial continuum of tissue injury rather than a simple binary pattern of cell death *versus* survival (Chu and Dupuy, 2014). Heat dissipates from the ablation center toward the periphery, generating gradients of thermal dose, oxygen tension, damage-associated molecular patterns (DAMPs), extracellular matrix (ECM) mechanics, nutrients, and immune cell infiltration. In this review, the sublethal thermal injury (STI) zone is operationally defined as the periablation region exposed to thermal doses insufficient to induce immediate and complete coagulative necrosis but sufficient to trigger cellular stress responses, delayed injury, vascular dysfunction, inflammatory recruitment, and matrix remodeling. This zone should not be regarded as a fixed temperature band, because biological outcomes depend on peak temperature, exposure duration, tissue perfusion, water content, cirrhosis-related fibrosis, and proximity to blood vessels. In general, temperatures above approximately 60 °C rapidly induce protein denaturation and coagulative necrosis, whereas lower temperature ranges, often around 42 °C–55 °C depending on exposure time, may elicit heat-shock responses, apoptosis or delayed cell death, endothelial injury, hypoxia, and stress-adaptive phenotypes in surviving cells.

Increasing evidence suggests that the STI zone may function as an active injury-repair microenvironment rather than a passive physical boundary. Within this region, necrotic and stressed cells release DAMPs such as high mobility group box 1, adenosine triphosphate, heat shock proteins, and lipid-rich debris, which recruit and activate myeloid cells (Krysko et al., 2012). Macrophages participate in phagocytosis of cellular debris and may acquire immunoregulatory phenotypes through phagocytosis-associated and metabolic programs (Liu et al., 2022). In parallel, sublethal heat, hypoxia, and altered ECM mechanics can activate hepatic stellate cells and promote their transition toward cancer-associated fibroblast-like states, contributing to collagen deposition, ECM stiffening, and mechanotransduction through Yes-associated protein/transcriptional coactivator with PDZ-binding motif (YAP/TAZ) and focal adhesion kinase-related pathways (Dupont et al., 2011). Vascular injury and hypoxia further stimulate vascular endothelial growth factor signaling and abnormal angiogenic repair (Carmeliet et al., 2011). Together, these processes may create a recurrence-permissive niche that supports residual tumor cell survival, epithelial–mesenchymal transition, stemness acquisition, invasion, and immune escape. The spatial and temporal organization of these multicellular interactions is summarized in Figure 1.

**FIGURE 1:**
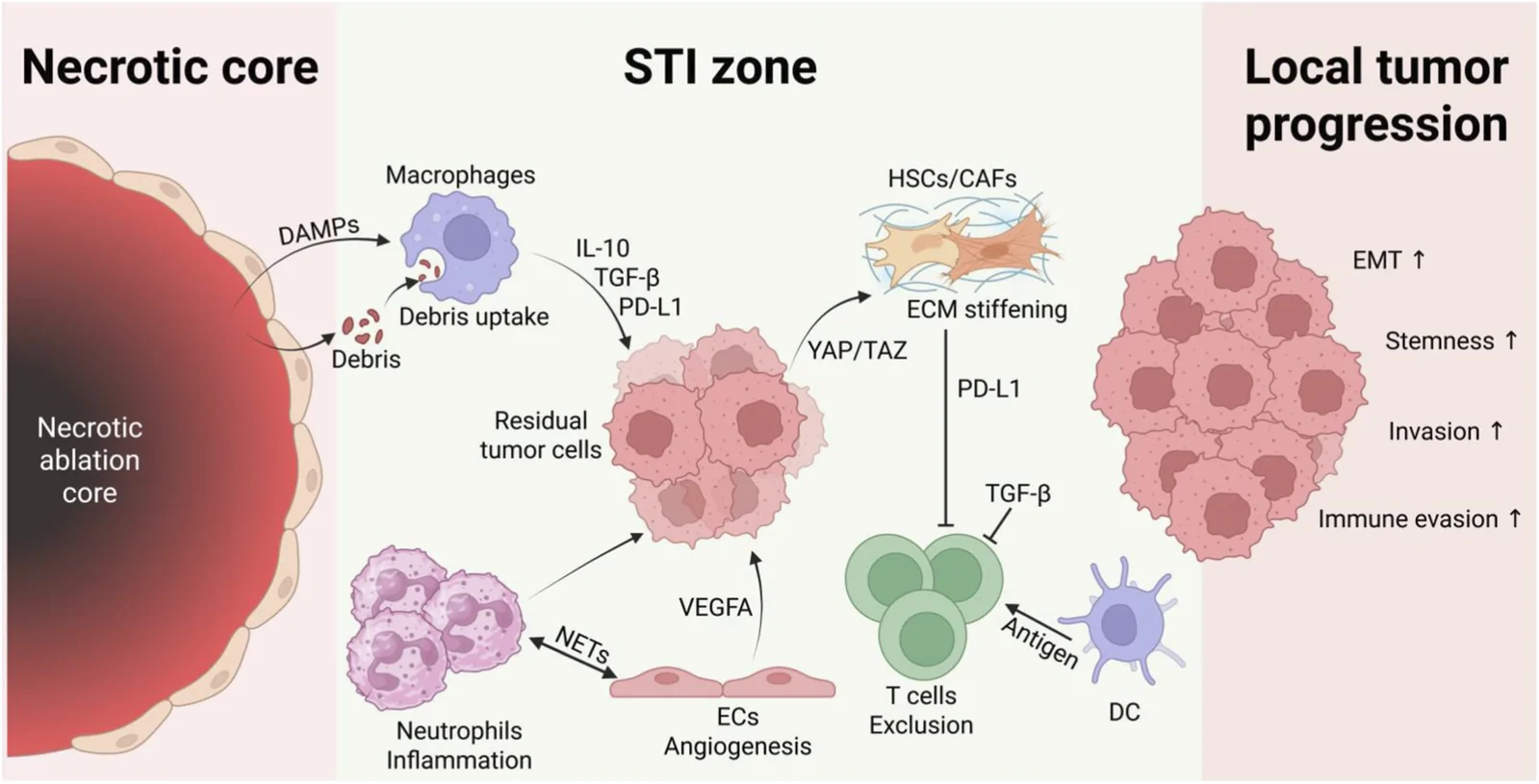
Spatial remodeling of the post-ablation multicellular recurrence niche in hepatocellular carcinoma. Thermal ablation generates a spatial injury continuum consisting of a necrotic core, a STI zone, and a region of local tumor progression. DAMPs and cellular debris released from the necrotic core diffuse into the STI zone, where they recruit and activate multiple immune, stromal, vascular, and residual tumor cell populations. Macrophages participate in debris uptake and may acquire immunosuppressive phenotypes; HSCs/CAFs remodel and stiffen the ECM; endothelial injury and hypoxia promote abnormal angiogenic repair; and neutrophils contribute to early inflammation, with potential NET-associated remodeling when inflammation persists. In parallel, antigen release may promote dendritic cell-mediated T-cell activation, whereas fibrotic matrix, abnormal vasculature, regulatory immune cells, and checkpoint signaling contribute to T-cell exclusion or dysfunction. Together, these interactions support residual tumor cell survival, EMT, stem-like plasticity, invasion, immune evasion, and local tumor progression. Solid arrows indicate mechanisms with stronger supporting evidence, whereas dashed arrows indicate inferred or proposed mechanisms. Abbreviations: CAFs, cancer-associated fibroblasts; DAMPs, damage-associated molecular patterns; ECM, extracellular matrix; EMT, epithelial–mesenchymal transition; HSCs, hepatic stellate cells; LTP, local tumor progression; VEGFA, vascular endothelial growth factor A.

However, the evidence supporting individual components of this model is heterogeneous. Some mechanisms have been demonstrated directly in HCC ablation models or clinical post-ablation specimens, whereas others are inferred from broader studies of HCC biology, tumor microenvironment (TME) remodeling, wound repair, or thermal injury. Therefore, this review distinguishes three levels of evidence: findings directly observed in post-ablation HCC settings; mechanisms extrapolated from related tumor or tissue-repair contexts; and engineering concepts proposed for future modeling. This distinction is essential to avoid presenting plausible but incompletely validated mechanisms as established conclusions.

Current experimental systems are not fully equipped to resolve this spatially organized and multicellular process. Conventional two-dimensional (2D) cultures are useful for testing defined molecular responses to heat stress but lack three-dimensional ECM architecture, oxygen and chemokine gradients, stromal mechanics, and immune–tumor interactions (Pampaloni et al., 2007). Animal models preserve systemic immunity, vascular structures, and liver-specific context, yet they are limited by variable thermal dose delivery, tissue opacity that restricts real-time imaging, interspecies differences in immune and stromal programs, high cost, and limited suitability for high-throughput sequential therapy testing (Zhang et al., 2019). These limitations have constrained mechanistic dissection of the ablation margin and delayed rational development of adjuvant strategies.

Engineered three-dimensional organoid-on-a-chip platforms may help bridge this methodological gap by integrating patient-derived HCC organoids, tunable ECM hydrogels, microfluidic control of thermal and hypoxic gradients, and selected stromal, myeloid, vascular, and immune components. In this review, we first discuss the STI zone as a spatially organized post-ablation niche in HCC. We then summarize multicellular remodeling involving myeloid cells, hepatic stellate cells/cancer-associated fibroblasts, endothelial cells, neutrophils, adaptive immune cells, and residual tumor cells. Finally, we evaluate organoid-on-a-chip systems as hypothesis-generating platforms for mechanistic modeling and personalized therapeutic screening, while emphasizing their current technical boundaries and translational challenges.

## 2 The ablation margin: a multicellular regenerative niche

### 2.1 Thermal ablation: beyond binary physical destruction

Thermal ablation does not simply divide tumor tissue into a necrotic core and a viable periphery. Instead, radiofrequency ablation, microwave ablation, and laser ablation generate a spatially continuous thermal dose gradient, in which biological outcomes are determined by peak temperature, exposure duration, tissue perfusion, water content, probe position, tumor geometry, and the heat-sink effect of adjacent vessels (Ahmed et al., 2011; Goldberg et al., 2000; Brace, 2011). Temperatures above approximately 60 °C generally induce rapid protein denaturation, membrane disruption, and coagulative necrosis, whereas lower but sustained thermal exposure may cause delayed cell death, vascular injury, stress adaptation, and inflammatory remodeling rather than immediate tissue destruction (Pillai et al., 2015; Chu and Dupuy, 2014).

### 2.2 Multidimensional gradients within the STI zone

The STI zone is shaped by multiple overlapping gradients beyond heat alone. Oxygen tension declines because of microvascular injury and impaired perfusion, leading to hypoxia-inducible factor-1α stabilization and downstream angiogenic and survival programs (Semenza, 2002). DAMPs, including high-mobility group box 1, extracellular adenosine triphosphate (ATP), and heat shock proteins, diffuse from necrotic and stressed cells and may activate innate immune responses (Krysko et al., 2012; Chen and Nuñez, 2010). However, persistent damage signaling may also sustain chronic inflammation and aberrant repair. In parallel, thermal injury alters ECM architecture through collagen contraction, denaturation, and cross-linking, thereby changing matrix stiffness and cellular mechanotransduction (Dupont et al., 2011; Paszek et al., 2005).

### 2.3 Stress adaptation of residual tumor cells

Residual tumor cells surviving within the STI zone are exposed to strong selective pressure. Heat shock proteins such as heat shock protein 70 (HSP70) and HSP90 support proteostasis after thermal stress (Mazurakova et al., 2023). Hypoxia-associated signaling may promote glycolytic adaptation, survival, and invasion, whereas matrix stiffening can activate integrin–focal adhesion kinase and YAP/TAZ (Semenza, 2012). STI stress has also been linked to epithelial–mesenchymal transition-like changes, including reduced epithelial adhesion and increased mesenchymal marker expression. Thus, the STI zone may function as a biologically active selection field in which residual tumor cells, stromal elements, vascular injury, and innate immune responses cooperate to create a recurrence-permissive niche (Liu et al., 2022).

## 3 Multicellular remodeling of the post-ablation TME

The ablation margin rapidly evolves from a transient thermal transition zone into a spatially organized wound-repair and tumor-supportive microenvironment. This process is highly time dependent. Within hours, DAMPs, complement activation, endothelial injury, and neutrophil recruitment dominate the local response. Over the following days, monocyte-derived macrophages accumulate and participate in cellular debris clearance, inflammatory resolution, and tissue remodeling. Subsequently, hepatic stellate cells (HSCs), cancer-associated fibroblast-like stromal cells (CAFs), and endothelial cells reshape the ECM, vascular architecture, and immune accessibility of the margin. As summarized in Figure 1, these events may convert the STI zone from a passive injury boundary into a multicellular niche that supports residual tumor cell survival, epithelial–mesenchymal transition (EMT), stem-like plasticity, immune evasion, and local tumor progression.

### 3.1 Macrophages: debris clearance and immunometabolic reprogramming

Macrophages are central regulators of the post-ablation TME. After thermal injury, DAMPs and chemokines promote the recruitment of circulating monocytes through axes such as CCL2/CCR2 and colony-stimulating factor 1/colony-stimulating factor 1 receptor (CSF-1/CSF1R) (Qian et al., 2011; Noy and Pollard Jeffrey, 2014). These macrophages initially contribute to debris clearance, including phagocytosis of apoptotic cells when present, necrotic fragments, membrane debris, and lipid-rich material released from damaged cells. Therefore, “cellular debris phagocytosis” is a more appropriate general term than efferocytosis unless apoptotic cell clearance has been specifically demonstrated.

Persistent debris uptake may impose metabolic stress on macrophages, including lipid accumulation, lysosomal burden, and altered cholesterol handling. In HCC radiofrequency ablation models, LC3-associated phagocytosis and PI3Kγ/AKT signaling have been implicated in post-ablation immunosuppression (Liu et al., 2022). Broader tumor and tissue-repair studies further suggest that lipid-responsive programs, including liver X receptor (LXR), peroxisome proliferator-activated receptor gamma (PPARγ), and TREM2-associated macrophage states, may contribute to macrophage remodeling, although their specific roles in post-ablation HCC remain incompletely validated (Molgora et al., 2020; Cheng et al., 2021). SPP1 should not be classified as a lipid-sensing pathway; rather, it is a secreted matricellular protein and a marker of specific macrophage subsets associated with ECM remodeling, angiogenesis, and immune suppression (Wei et al., 2018). Through secretion of interleukin-10 (IL-10), transforming growth factor-beta (TGF-β), vascular endothelial growth factor A (VEGFA), and immune checkpoint ligands such as programmed death-ligand 1 (PD-L1), macrophages may create a permissive niche for residual tumor cells to survive thermal stress and acquire invasive phenotypes (Mantovani et al., 2017).

### 3.2 HSCs/CAFs: ECM remodeling and stiffness-driven invasion

HSCs are major stromal effectors in the injured liver. Following ablation, inflammatory cytokines, hypoxia, and thermally altered ECM may activate HSCs toward α-SMA-positive myofibroblastic or CAF-like states (Kisseleva and Brenner, 2021; Affo et al., 2017). Thermal injury can induce collagen denaturation, contraction, and cross-linking, increasing matrix stiffness at the ablation margin. Although direct quantitative stiffness data in post-ablation human HCC remain limited, general mechanobiology studies support the concept that stiffened ECM activates integrin–focal adhesion kinase (FAK) signaling and YAP/TAZ mechanotransduction in tumor and stromal cells (Dupont et al., 2011; Paszek et al., 2005).

Activated stromal cells may deposit collagen I, collagen III, fibronectin, periostin, and other ECM components, thereby generating a dense matrix that promotes tumor cell migration and restricts immune cell penetration (Sahai et al., 2020; Kalluri, 2016). Matrix metalloproteinases (MMPs), including MMP2 and MMP9, may further remodel basement membrane structures and release matrix-bound growth factors (Kessenbrock et al., 2010). Importantly, CAF-like stromal cells and macrophages can reinforce each other: stromal-derived CCL2 and CSF-1 promote myeloid recruitment, whereas macrophage-derived TGF-β and platelet-derived growth factor (PDGF) support fibroblast activation (Mao et al., 2021). This reciprocal loop may contribute to a fibrotic, immune-excluded ablation margin. Accordingly, targeting TGF-β, FAK, or CSF1R represents a rational but still investigational strategy that requires careful timing to avoid impairing physiological liver repair.

### 3.3 Endothelial cells: hypoxia, angiogenesis, and barrier dysfunction

Thermal ablation damages sinusoidal and microvascular structures at the tumor margin, resulting in hypoperfusion, tissue hypoxia, and stabilization of hypoxia-inducible factor-1 alpha (HIF-1α). Hypoxia can promote VEGFA, angiopoietin-2 (ANG2), and fibroblast growth factor 2 (FGF2) production by residual tumor cells, macrophages, and stromal cells, thereby stimulating angiogenic sprouting (Semenza, 2002; Ferrara et al., 2003). However, newly formed vessels in tumors are often immature, tortuous, and hyperpermeable, with defective pericyte coverage and disrupted endothelial junctions (Jain, 2005). Such abnormal vasculature may sustain minimal nutrient delivery to residual tumor cells while failing to restore uniform oxygenation.

Endothelial dysfunction also shapes immune access. Reduced expression of adhesion molecules, altered chemokine gradients, increased vascular permeability, and elevated interstitial pressure may limit cytotoxic T-cell extravasation and drug delivery (Motz et al., 2014). Therefore, vascular normalization after ablation may have dual benefits: improving perfusion and enhancing immune infiltration. Nevertheless, anti-angiogenic therapy must be considered cautiously in cirrhotic livers, where vascular repair and hepatic regeneration are clinically relevant concerns.

### 3.4 Neutrophils: early repair and potential pro-recurrence inflammation

Neutrophils are among the earliest leukocytes recruited after thermal injury. DAMPs, complement fragments, and chemokines such as CXCL1, CXCL2, and CXCL8 can drive rapid neutrophil influx into the injured margin (Kolaczkowska and Kubes, 2013). In the early phase, neutrophils contribute to antimicrobial defense, debris processing, and initial matrix remodeling through reactive oxygen species, antimicrobial peptides, and proteases (Pham, 2006). These functions may support sterile wound repair.

However, persistent or excessive neutrophil activity can aggravate tissue injury. Neutrophil elastase, myeloperoxidase, and reactive oxygen species may damage endothelial barriers and amplify local inflammation (Coffelt et al., 2016). In cancer models, neutrophils can also promote angiogenesis and tumor progression through MMP9-mediated VEGF release, immunosuppressive granulocytic myeloid-derived suppressor cell-like states, and neutrophil extracellular trap (NET)-associated mechanisms (Coffelt et al., 2016; Nozawa et al., 2006; Demers et al., 2016). Direct clinical evidence for NETosis in the post-ablation HCC margin remains limited; thus, NET-associated recurrence should be presented as a plausible mechanism extrapolated from broader cancer and inflammation studies rather than an established post-ablation fact. Spatial and temporal control of neutrophil responses may therefore be important: early repair-promoting functions should be preserved, whereas chronic neutrophilic inflammation and NET-associated remodeling may need to be restrained.

### 3.5 Adaptive immune cells: antigen release, T-Cell exclusion, and dysfunction

Thermal ablation can release tumor antigens, heat-shock protein-associated peptides, and inflammatory signals that may facilitate dendritic cell (DC) uptake, cross-presentation, and tumor-specific T-cell priming (den Brok et al., 2004). In HCC, radiofrequency ablation has been reported to enhance tumor-associated antigen-specific T-cell responses in some patients, supporting the concept of an *in situ* vaccination effect (Mizukoshi et al., 2013). However, this response is often transient and may be insufficient to prevent local tumor progression.

Several suppressive mechanisms may limit adaptive immunity at the ablation margin. Hypoxia and inflammatory chemokines can favor regulatory T-cell recruitment and function, while macrophage-derived PD-L1, IL-10, and TGF-β can attenuate cytotoxic T-cell activity (Mantovani et al., 2017; Dikiy and Rudensky, 2023). Chronic antigen exposure and checkpoint signaling may contribute to T-cell exhaustion, characterized by increased expression of inhibitory receptors such as programmed cell death protein 1 (PD-1), T-cell immunoglobulin and mucin domain-containing protein 3 (TIM-3), and lymphocyte-activation gene 3 (LAG-3), together with reduced effector cytokine and granzyme production (Wherry, 2011). In addition, CAF-rich ECM and abnormal vasculature may physically and functionally restrict T-cell infiltration. These barriers help explain why antigen release after ablation does not consistently translate into durable antitumor immunity. Consequently, effective post-ablation immunotherapy may require not only checkpoint blockade, but also coordinated remodeling of myeloid, stromal, and vascular barriers. Importantly, experimental models designed to evaluate T-cell infiltration, checkpoint dynamics, or anti-PD-1 responsiveness must include autologous tumor-reactive T cells or tumor-infiltrating lymphocytes; DCs are additionally required if antigen presentation and T-cell priming are to be modeled.

## 4 Methodological challenges and limitations of current research frameworks

Although post-ablation TME remodeling has been investigated using both *in vitro* and *in vivo* systems, current models still have difficulty capturing the spatially organized and temporally evolving nature of the ablation margin. The key challenge is not simply to determine whether residual tumor cells survive thermal stress, but to understand how thermal gradients, hypoxia, ECM remodeling, vascular injury, and immune-cell recruitment interact to promote local tumor progression. Existing approaches therefore face two major limitations: the lack of spatial fidelity in reductionist *in vitro* systems and the limited controllability of complex animal models.

### 4.1 2D cell culture: molecular simplicity but limited spatial fidelity

Traditional 2D monolayer cultures remain widely used in thermal injury and cancer biology because they are simple, inexpensive, reproducible, and suitable for testing defined molecular responses. In typical ablation-related experiments, tumor cell lines or primary cells are exposed to uniform heat stress for a defined duration, followed by assessment of viability, apoptosis, heat-shock protein expression, migration, or epithelial–mesenchymal transition-related markers. Such systems are useful for identifying cell-intrinsic stress-response pathways and for preliminary drug testing.

However, 2D cultures poorly recapitulate the biological architecture of the post-ablation margin. In clinical ablation, cells are not exposed to a single homogeneous temperature; rather, they are distributed along a continuous thermal-dose gradient extending from the necrotic core to the STI zone and distal viable tissue (Chu and Dupuy, 2014; Ahmed et al., 2011). In addition, 2D cultures lack the three-dimensional ECM architecture that regulates tumor-cell polarity, migration, mechanotransduction, and drug response (Pampaloni et al., 2007; Kolenda et al., 2016). They also fail to reproduce oxygen and nutrient gradients, matrix stiffening after thermal collagen remodeling, vascular injury, and immune-cell trafficking. Most importantly, conventional 2D systems cannot model spatially restricted interactions among residual tumor cells, macrophages, HSCs, endothelial cells, neutrophils, and T cells. As a result, mechanisms identified in 2D cultures may reflect isolated thermal tolerance rather than the multicellular recurrence niche that develops after ablation *in vivo*.

### 4.2 Animal models: physiological complexity but limited experimental control

Animal models, including subcutaneous xenografts, chemically induced liver tumors, genetically engineered models, and orthotopic HCC models in mice, rats, or rabbits, remain indispensable for studying ablation biology. These models preserve vascular perfusion, stromal architecture, systemic inflammation, and organ-level immune responses, making them especially valuable for evaluating ablation combined with immunotherapy or anti-angiogenic therapy (Zhang et al., 2019; Lee et al., 2014).

Nevertheless, animal models have important limitations. First, precise control of thermal dose is difficult *in vivo*. Even under identical ablation settings, tumor size, tissue hydration, cirrhosis-related fibrosis, probe position, and local blood flow can generate heterogeneous temperature distributions and inconsistent ablation margins. Second, real-time visualization of multicellular remodeling at high spatial and temporal resolution remains challenging because of tissue opacity and motion artifacts. Dynamic processes such as macrophage recruitment, phagocytosis of cellular debris, endothelial sprouting, and fibroblast-mediated ECM contraction are therefore usually inferred from endpoint histology rather than directly observed. Third, animal studies are expensive, low-throughput, and poorly suited for systematically optimizing the timing, sequence, and dose of post-ablation combination therapies. Finally, interspecies differences in macrophage metabolism, T-cell exhaustion, stromal activation, and cirrhosis-associated liver repair limit direct extrapolation from murine models to human HCC. These limitations underscore the need for complementary human-relevant platforms that can preserve multicellular complexity while allowing precise spatial control of thermal, hypoxic, and mechanical cues.

## 5 Organoid-on-a-chip platforms: a new paradigm for reconstructing the ablation margin

Engineered organoid-on-a-chip systems provide an emerging, but still developing, strategy to model the spatial and multicellular complexity of the post-ablation HCC margin *in vitro*. Unlike conventional 2D cultures, these platforms can integrate three-dimensional tumor architecture, matrix mechanics, soluble gradients, fluidic control, and live imaging within a miniaturized system (Ingber, 2022). In the context of post-ablation recurrence, their major value lies not in fully replacing animal models, but in enabling controlled, reductionist reconstruction of selected components of the necrotic core–STI zone–distal viable tissue axis. As illustrated in Figure 2, we propose an HCC-focused organoid-on-a-chip framework composed of patient-derived tumor organoids, tunable ECM hydrogels, microfluidic control of physical gradients, and defined multicellular co-culture modules.

**FIGURE 2:**
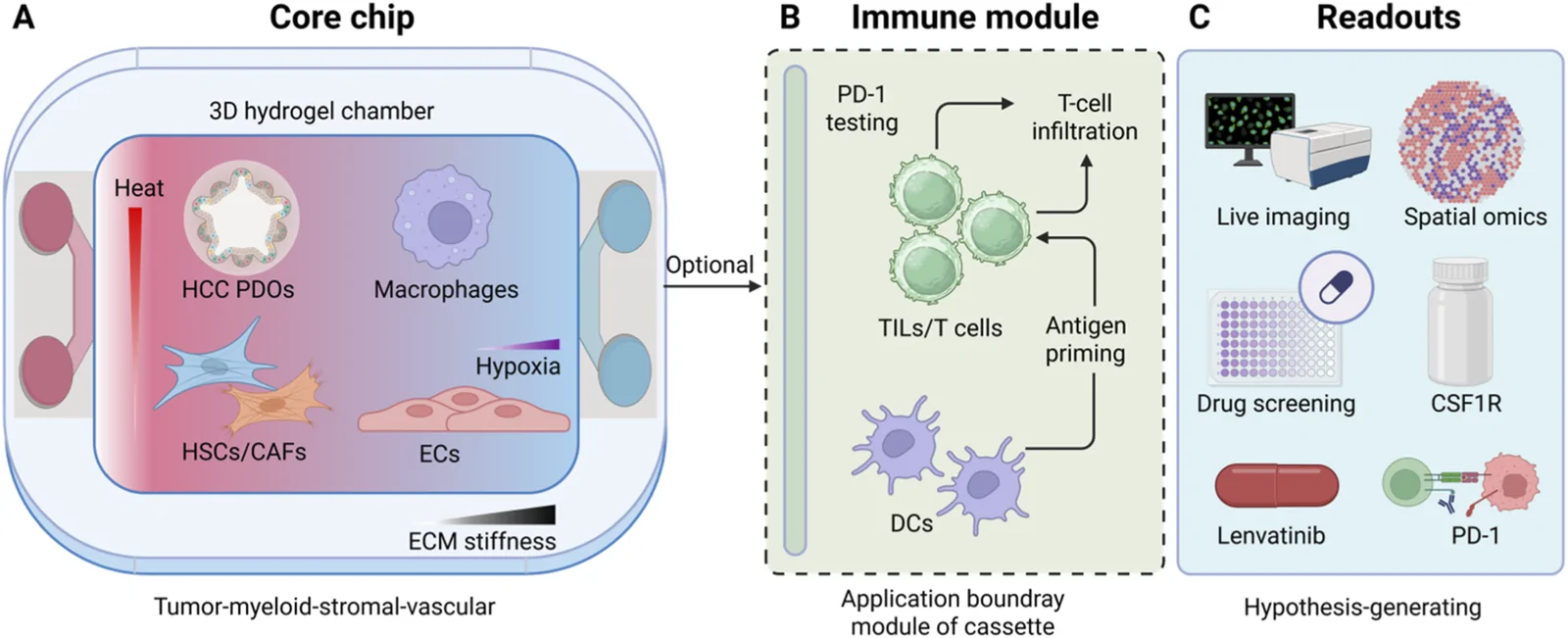
HCC organoid-on-a-chip framework for modeling the post-ablation recurrence niche. **(A)** Core tumor–myeloid–stromal–vascular module. Patient-derived HCC organoids are co-cultured with macrophages, HSCs/CAFs, and ECs within a tunable three-dimensional hydrogel chamber. Controlled thermal exposure, hypoxia, and ECM stiffness are intended to reproduce selected features of the ablation margin and enable investigation of residual tumor cell behavior and multicellular remodeling. **(B)** Optional immune-reconstitution module. Autologous TILs or tumor-reactive T cells are required for studies of T-cell infiltration and responses to PD-1 blockade. Incorporating DCs additionally enables investigation of antigen presentation and T-cell priming. These immune-dependent applications cannot be adequately evaluated using the core module alone. **(C)** Proposed analytical and drug-screening applications. Live imaging and spatial omics provide complementary approaches to characterizing cellular responses. Candidate interventions for exploratory testing include CSF1R inhibition, lenvatinib, and PD-1 blockade, with the latter requiring the immune-reconstituted platform. Studies of treatment combinations and sequencing should consider both antitumor effects and potential interference with liver repair. The schematic represents a conceptual, hypothesis-generating framework rather than a fully validated post-ablation model or an established therapeutic regimen. Abbreviations: CAFs, cancer-associated fibroblasts; CSF1R, colony-stimulating factor 1 receptor; DCs, dendritic cells; ECM, extracellular matrix; ECs, endothelial cells; HCC, hepatocellular carcinoma; HSCs, hepatic stellate cells; PDOs, patient-derived organoids; PD-1, programmed cell death protein 1; TILs, tumor-infiltrating lymphocytes.

### 5.1 Patient-derived HCC organoids: preserving tumor heterogeneity

PDOs from HCC specimens can preserve key histological, genetic, and interpatient drug-response features of primary liver tumors, making them more clinically relevant than long-established cell lines for modeling residual tumor cell behavior (Li et al., 2019). In a post-ablation setting, HCC PDOs could be used to assess how sublethal heat exposure, hypoxia, and altered matrix mechanics influence stress adaptation, epithelial–mesenchymal transition, stem-like plasticity, and therapeutic vulnerability. Paired pre- and post-ablation or primary and recurrent PDO models, when available, may further help identify recurrence-associated phenotypes. However, these applications should be interpreted cautiously because PDO establishment efficiency, culture duration, and selective outgrowth can influence the fidelity of patient representation.

### 5.2 Tunable matrix hydrogels: modeling mechanical remodeling

Thermal injury can alter ECM architecture through collagen contraction, denaturation, and remodeling, thereby changing the mechanical cues sensed by residual tumor and stromal cells. Tunable hydrogels, including gelatin methacryloyl (GelMA), collagen-based matrices, and composite hydrogels, offer a practical way to model these biomechanical changes. By adjusting polymer concentration, crosslinking density, and matrix composition, stiffness gradients can be generated to approximate the transition from softer liver-like tissue to a fibrotic periablation margin. Such systems are particularly useful for studying integrin–FAK–YAP/TAZ mechanotransduction, a pathway broadly implicated in stiffness-driven tumor progression and stromal activation (Virdi and Pethe, 2021). Nevertheless, hydrogel stiffness should be viewed as a simplified surrogate for post-ablation ECM remodeling, not a complete reconstruction of the native cirrhotic and thermally injured liver matrix.

### 5.3 Microfluidic thermal and hypoxic control: reconstructing spatial gradients

Microfluidic devices can provide spatial control over temperature, oxygen tension, soluble mediators, and fluid flow, making them attractive for modeling the ablation margin. A proposed post-ablation chip may include programmable microheaters to generate localized thermal injury, oxygen-controlled channels to reproduce hypoxic stress, and laminar-flow gradient systems to expose cells to DAMPs such as HMGB1 or ATP. These modules would allow investigators to test how thermal dose, hypoxia, and inflammatory gradients jointly shape residual tumor cell survival and stromal remodeling. Importantly, most such configurations remain conceptual or partially validated in related organ-on-a-chip systems rather than fully established for post-ablation HCC. Therefore, claims regarding precise reconstruction of the complete ablation margin should be framed as a future engineering goal.

### 5.4 Multicellular co-culture and therapeutic screening

The core version of the proposed platform would include HCC PDOs, macrophages, HSCs, and endothelial cells. This four-component model is suitable for studying tumor–myeloid–stromal–vascular interactions, including macrophage migration and debris clearance, HSC activation, ECM remodeling, endothelial sprouting, and paracrine support of residual tumor cells (Goldrick et al., 2023; Mun et al., 2022). Advanced imaging, multiplex immunofluorescence, live-cell tracking, and spatial transcriptomic readouts could further capture dynamic remodeling events at single-cell or spatially resolved levels.

However, the application boundary of this core platform must be clearly defined. A model containing only PDOs, macrophages, HSCs, and endothelial cells is not sufficient to evaluate T-cell infiltration, immune checkpoint dynamics, or anti-PD-1 responsiveness. These applications would require an expanded immune-competent platform incorporating autologous tumor-infiltrating lymphocytes or tumor-reactive T cells. If the aim is to model post-ablation antigen presentation and T-cell priming, dendritic cells should be added as an additional module. Thus, anti-PD-1 testing should be considered feasible only in appropriately immune-reconstituted systems.

Similarly, sequential regimens combining ablation with CSF1R inhibition, anti-angiogenic therapy such as lenvatinib, and PD-1 blockade should be presented as hypothesis-generating rather than clinically established. Their timing requires careful optimization because macrophages, angiogenic pathways, and stromal repair programs also contribute to debris clearance, wound healing, vascular restoration, liver regeneration, and maintenance of hepatic reserve in cirrhotic patients. Future chip-based screening should therefore evaluate not only antitumor efficacy, but also liver-specific safety windows and repair-related toxicity.

## 6 Conclusion

Local tumor progression remains a major obstacle to durable disease control after thermal ablation for HCC. This review highlights the STI zone as a spatially organized and biologically active periablation niche rather than a simple boundary of incomplete tumor destruction. This niche is shaped by overlapping thermal-dose, hypoxic, mechanical, inflammatory, and immune gradients. Within this region, residual tumor cells may acquire stress-adaptive phenotypes, while macrophages participate in phagocytosis of apoptotic cells and cellular debris and may undergo phagocytosis-associated immunometabolic reprogramming. HSCs can be activated into α-SMA-positive CAFs within a stiffened ECM, promoting matrix remodeling and YAP/TAZ-associated mechanotransduction. In parallel, vascular injury and abnormal angiogenesis may aggravate hypoxic heterogeneity and limit effective immune cell trafficking. Neutrophil-associated inflammation, possible NET-related injury, regulatory immune recruitment, T-cell exclusion, and checkpoint-mediated dysfunction may further contribute to an immunosuppressive environment that favors residual tumor survival and local regrowth.

Conventional 2D cultures and animal models remain indispensable but have important limitations in reproducing spatial thermal gradients, matrix mechanics, hypoxia, and dynamic multicellular interactions. Engineered organoid-on-a-chip systems offer a promising, although still developing, strategy to model selected features of the post-ablation HCC microenvironment. A core platform incorporating PDOs, macrophages, HSCs, endothelial cells, tunable GelMA-based matrices, and microfluidic thermal or oxygen control may help interrogate tumor–myeloid–stromal–vascular crosstalk. However, applications involving T-cell infiltration, immune checkpoint dynamics, or anti-PD-1 responsiveness require immune-competent expansion with autologous tumor-infiltrating lymphocytes or tumor-reactive T cells, while dendritic cells are needed to model antigen presentation and T-cell priming.

Future work should define the evidence hierarchy more rigorously by distinguishing mechanisms directly demonstrated in post-ablation HCC from those extrapolated from broader TME or wound-healing studies. Sequential strategies combining ablation with macrophage modulation, anti-angiogenic therapy, or immune checkpoint blockade should be regarded as hypothesis-generating and must be optimized with attention to liver regeneration, wound healing, vascular repair, and cirrhotic hepatic reserve. Despite practical barriers, including variable organoid establishment, prolonged culture time, high cost, limited access to autologous immune cells, and standardization challenges, multicellular organoid-on-a-chip models may provide a valuable translational framework for mechanism-guided and safety-conscious personalization of post-ablation therapy in HCC.
